# An Improved SHANEL Procedure for Clearing and Staining Brain Tissue from Multiple Species

**DOI:** 10.3390/ijms26083569

**Published:** 2025-04-10

**Authors:** Fu Zeng, Lian Huang, Ding Han, Renjie Liu, Kongjia Zhang, Yuwen Su, Tong Su, Yarong Lin, Jianbo Xiu

**Affiliations:** 1 State Key Laboratory of Common Mechanism Research for Major Diseases, Institute of Basic Medical Sciences, Chinese Academy of Medical Sciences, School of Basic Medicine Peking Union Medical College, Beijing 100005, China; zf316125@163.com (F.Z.); huanglian@ibms.pumc.edu.cn (L.H.); regina241009@163.com (R.L.); zkj0012@163.com (K.Z.); suez19215@163.com (Y.S.); 13678917505@126.com (T.S.); linaong@163.com (Y.L.); 2 Neuroscience Center, Chinese Academy of Medical Sciences, Beijing 100005, China; 3 School of Basic Medical Sciences, Capital Medical University, Beijing 100069, China; hdyhnn2025@163.com; 4 State Key Laboratory of Complex, Severe, and Rare Diseases, Beijing 100005, China

**Keywords:** tissue clearing, multiple species, autofluorescence

## Abstract

Recent advances in tissue clearing chemistry have revolutionized three-dimensional imaging by enabling whole-organ antibody labeling, even in thick human tissue samples. However, these techniques face limitations, including reduced clearance efficiency in thick tissue blocks, prolonged processing times, and other challenges specific to formalin-fixed human brain tissue, such as autofluorescence due to the presence of lipofuscin, neuromelanin pigments, and residual blood. To address these challenges, we have developed an improved SHANEL procedure, iSHANEL, which is compatible with brain tissue from multiple species, including nonrodents. iSHANEL significantly enhances the tissue transparency. It effectively removes lipids, particularly sphingolipids, from brain tissue across different species. For brain vasculature imaging, it offers the better visualization of vascular structures at high magnification, reducing light scattering and background noise. Moreover, iSHANEL can be effectively applied to large tissue from pigs and cynomolgus monkeys, and it preserves protein immunogenicity well, as evidenced by immunostaining with the microglia-specific marker IBA-1. We also investigated methods to reduce blood vessel autofluorescence. Overall, iSHANEL provides an improved procedure for the high-resolution 3D imaging of brain tissue across multiple species.

## 1. Introduction

The earliest attempts at tissue clearing date back to 1911, when the German anatomist Werner Spalteholz employed wintergreen oil and benzyl benzoate to render cardiac tissue transparent, enabling the visualization of the heart’s vascular system. Most tissue clearing methods involve the removal of lipids and water, replacing them with a medium with a higher refractive index (RI). This process leads to a more uniform RI across the tissue and reduces light scattering, ultimately enhancing the imaging quality [1]. Over the past decade, tissue clearing technology has gained significant attention in the field of neurobiology. This technique, when combined with fluorescence microscopy, provides an efficient approach to visualizing three-dimensional tissue structures, circumventing the cumbersome and time-consuming process of reconstructing serial thin sections [2]. Light-sheet fluorescence microscopy is a powerful technique that enables rapid imaging with minimal phototoxicity, making it ideal for the volumetric imaging of biological samples [3]. In this study, we used a light-sheet microscope, which supports the imaging of samples ranging from the micrometer to centimeter scale, with a resolution down to the sub-micron level.

Currently, tissue clearing techniques can be categorized into three main approaches, hydrophobic, hydrophilic, and hydrogel-based methods, each with its own advantages and limitations [2]. Hydrophilic methods are widely used due to their good biocompatibility and low toxicity; however, they have relatively low clearing efficiency and require long processing times, making them more suitable for small-volume samples [4]. Nevertheless, when combined with tissue expansion techniques, hydrophilic methods can enhance the imaging resolution to some extent [5]. In contrast, hydrophobic methods allow for the rapid and efficient clearing of large-volume tissue but often involve chemical reagents with high toxicity, posing potential safety risks [6]. Hydrogel-based methods create a stable network within the tissue to immobilize biomolecules, thereby enabling uniform lipid removal while minimizing structural damage and molecular loss. However, these methods involve complex procedures, are costly, and typically require specialized equipment, limiting their widespread application [3].

Despite significant advancements in clearing techniques, particularly in terms of achieving transparency and the effective immunofluorescence imaging of rodent brain tissue [7,8,9], their application to human brains remains challenging [3,10,11]. First, the high content of lipofuscin and the retention of blood (due to the inability to perfuse the tissue) result in strong autofluorescence [12,13]. Second, the high density of neurons and myelin, coupled with the greater variability in postmortem tissue conditions, significantly impacts the clearance efficiency. Furthermore, prolonged formalin fixation causes pH shifts and tissue oxidation, leading to antigen epitope masking and damage, which compromise the immunolabeling quality [14]. Finally, unlike rodents, human brains cannot be genetically engineered to express fluorescent proteins, which significantly complicates protein imaging.

To address the challenges outlined above, we introduce the iSHANEL technique, which extends conventional experimental models from rodents to pigs and monkeys. This approach utilizes both antibodies and dyes to label the protein and whole-brain vasculature, respectively. Additionally, we compare the effects of eliminating vascular autofluorescence using three different reagents and active perfusion. More importantly, for the first time, our study examines changes in lipid content across various clearing methods through lipid metabolism, providing valuable insights for the future development of novel lipid removal reagents. In summary, we present a versatile brain tissue clearing technique that is applicable across multiple species, capable of eliminating vascular autofluorescence, and compatible with a range of staining methods.

## 2. Results

### 2.1. Comparison of Different Methods for Clearance of Large Brain Tissue

**Figure 1:**
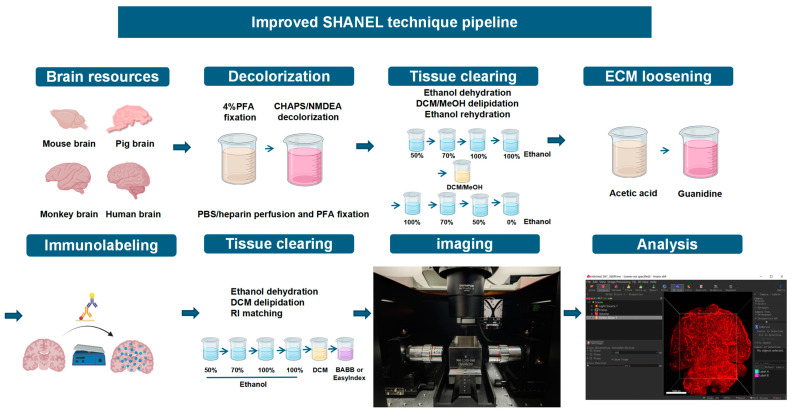
Overview of the iSHANEL pipeline.

**Figure 2:**
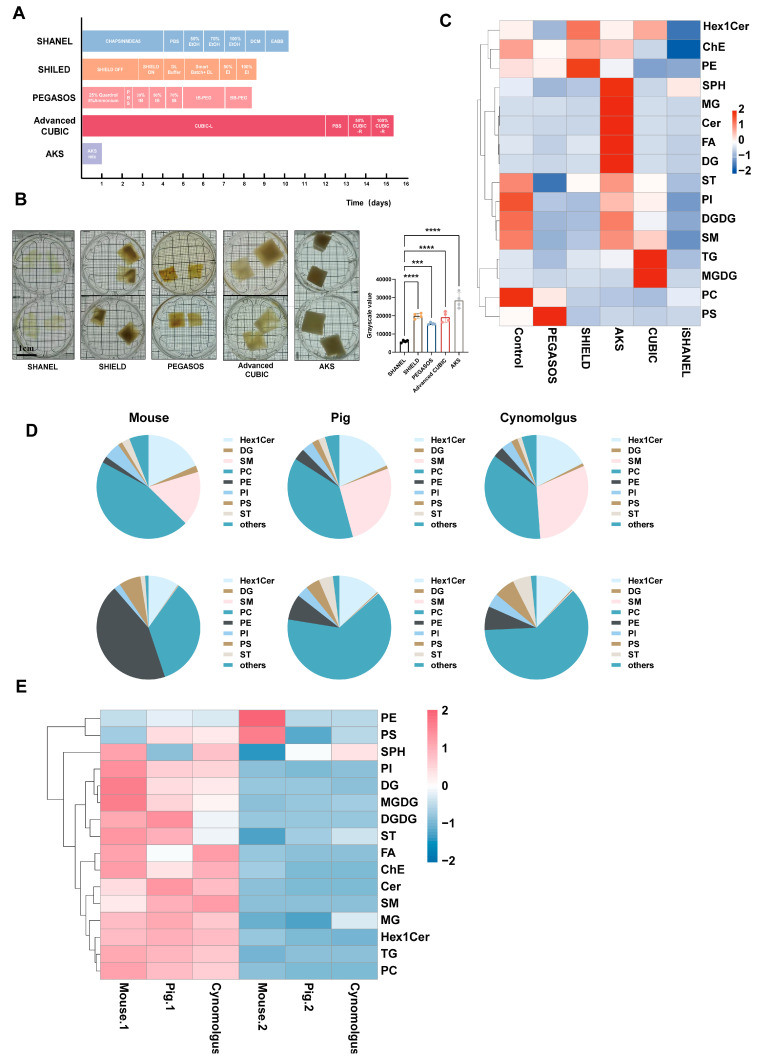

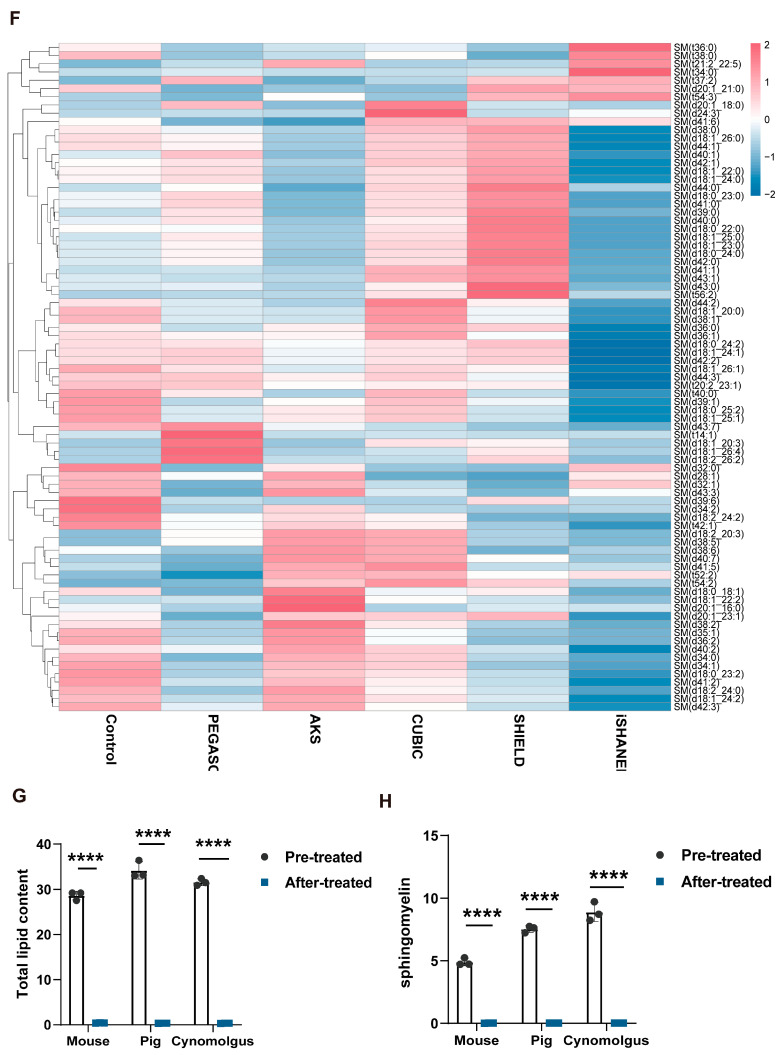
iSHANEL technology effectively removes sphingolipids from large brain tissue samples. (**A**) Timelines of the different tissue transparency methods. (**B**) Transparency effectiveness and gray value statistical analysis of 2.5 cm^3^ pig brain tissue samples treated with different transparency methods (n = 4/group, *** *p* < 0.005, **** *p* < 0.0001; one-way ANOVA with Tukey’s multiple comparison test). Scale bars, 1 cm. (**C**) Heatmap showing the major changes in lipid content before and after treatment with different transparency methods. (**D**) Pie charts showing the changes in the proportions of major lipid types in brain tissue from different species before and after iSHANEL treatment. (**E**) Heatmap showing the changes in the content of different types of lipids in mouse, pig, and monkey brains after iSHANEL treatment. (**F**) Changes in sphingolipid content in brain tissue from different species treated with different methods. (**G**) Changes in total the lipid content in mouse, pig, and cynomolgus monkey brains before and after iSHANEL treatment (n = 3/group, **** *p* < 0.0001, unpaired two-tailed *t* tests with Welch’s correction). (**H**) Changes in the total sphingomyelin content in mouse, pig, and cynomolgus monkey brains before and after iSHANEL treatment (n = 3/group, **** *p* < 0.0001, unpaired two-tailed *t* tests with Welch’s correction).

The following schematic diagram illustrates the methodological steps of this experiment, providing a clear overview of the experimental procedures (Figure 1). To evaluate the effects of the current mainstream tissue clearing technologies for large brain tissue (Figure 2A), we tested five approaches, namely SHANEL, SHIELD, PEGASOS, Advanced CUBIC, and AKS, in pig brain tissue sections with an average volume of 2.5 cm^3^. These five techniques were selected as representative examples of three main types of methods: hydrophobic, hydrophilic, and hydrogel-based [15,16]. SHANEL and PEGASOS are hydrophobic-based, Advanced CUBIC is hydrophilic-based, and SHIELD is hydrogel-based. Additionally, AKS was included because of its rapid one-step procedure, which significantly reduces the processing time. The results showed that, compared with other transparency technologies, SHANEL has the best effect and can render tissue up to 1 cm deep transparent (Figure 2B). Therefore, we developed an improved SHANEL technology, iSHANEL, with better transparency effects than the SHANEL technology. The difficulty in making brain tissue transparent lies in removing a large amount of the lipids present in the myelin sheath, which is composed of a high proportion of lipids [17]. These lipids strongly scatter light, which hinders light penetration and affects tissue transparency. Sphingolipids account for approximately 20–30% of the total lipids in the adult brain. To quantitatively evaluate the effects of different transparency reagents to scavenge lipids (represented by sphingolipids), we performed a targeted lipidomics analysis on brain tissue samples from pig brains before and after treatment. The results indicated that, compared with other tissue clearing techniques, iSHANEL resulted in better lipid removal from brain tissue (Figure 2C). To assess the efficacy of iSHANEL in enhancing lipid clearance from brain tissue across different species, we conducted untargeted lipidomics analyses before and after iSHANEL treatment. The findings demonstrated that iSHANEL effectively removed sphingomyelins regardless of their initial abundance, achieving high clearance efficiency in brain tissue with elevated sphingomyelin levels, such as that of cynomolgus monkeys and pigs, as well as in mouse brain tissue with lower sphingomyelin content (Figure 2D–H).

### 2.2. Clearing Brain Tissue with iSHANEL and 3D Imaging

**Figure 3:**
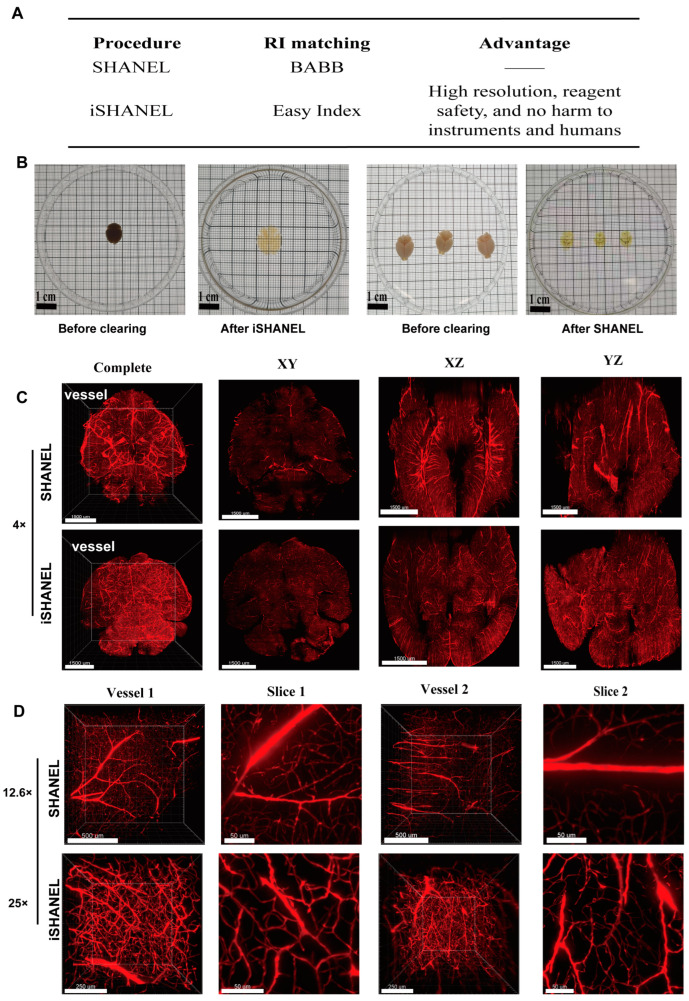
iSHANEL technology effectively enhances tissue transparency and improves imaging resolution. (**A**) The difference between iSHANEL technology and SHANEL technology. (**B**) iSHANEL technology and SHANEL technology were used to transparentize whole mouse brains. Scale bars, 1 cm. (**C**) Brain blood vessels labeled with lysine-fixable dextran conjugated with tetramethylrhodamine (red) after SHANEL treatment. Low-magnification image (4×) showing the vascular network. Scale bars, 1,500 um. (**D**) High-magnification image (25×, 12.6×) revealing the fine details and clarity of the blood vessels. Vessel scale bars: 500 μm at 12.6× magnification, 250 μm at 25× magnification. Slice scale bars: 50 μm.

The current tissue clearing techniques still have several limitations, including the small size of the stained samples, lengthy experimental procedures, insufficient staining depths, and limited species compatibility. In particular, hydrophobic-based clearing methods face challenges such as potential damage to sample integrity and risks to human safety due to toxic reagents [18]. Compared with SHANEL, iSHANEL expands the range of compatible dyes for staining, enhances the imaging resolution, and improves the cross-species compatibility (Figure 3A). To evaluate the efficacy of iSHANEL, we first applied this technique to mouse brains (Figure 3B), and iSHANEL demonstrated superior full-brain transparency in mice. Compared with SHANEL, iSHANEL results in less tissue yellowing, producing clearer and more neutral coloration. In contrast, SHANEL exhibited a more pronounced yellowing effect. Additionally, owing to its corrosive properties, BABB is limited to short-term observations in culture dishes. Furthermore, the brain vasculature was labeled with the vascular dye tetramethylrhodamine, after which the tissue was cleared with iSHANEL and SHANEL. At low magnification (4×), iSHANEL and SHANEL exhibited comparable tissue clearing performance, as both effectively preserved the overall structural integrity of the tissue (Figure 3C). However, at high magnification (25×), iSHANEL demonstrated significant advantages (Figure 3D). Compared with SHANEL, iSHANEL provides the superior visualization of vascular structures, including branch delineation, continuity, and overall clarity, in 100-μm-thick tissue sections. Furthermore, iSHANEL reduces light scattering and background noise during high-resolution imaging, enhancing the visualization of the fine tissue structures. These findings highlight the superior performance of iSHANEL in high-precision three-dimensional imaging, offering a more reliable technique for detailed histological analysis.

### 2.3. Application of iSHANEL Technology to Brain Tissue from Multiple Species

**Figure 4:**
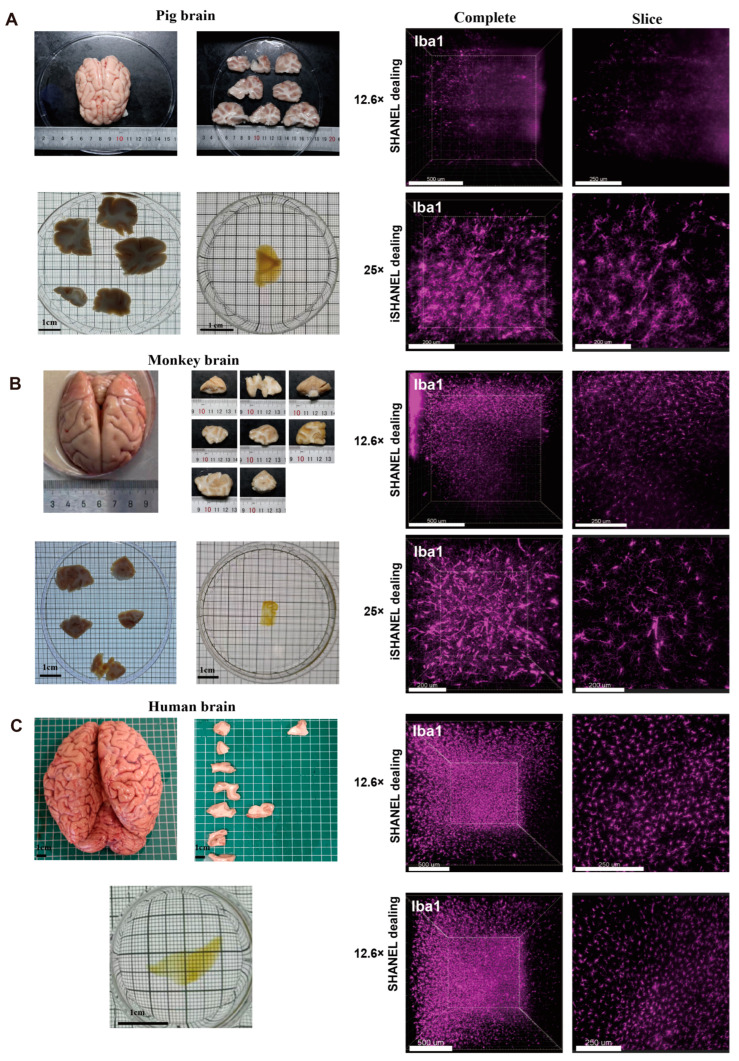
iSHANEL technology can be effectively applied to brain tissue from multiple species. (**A**) The transparency effect of SHANEL and iSHANEL on pig brain tissue and subsequent staining with the microglia-specific marker IBA1 (purple). At 12.6× magnification, the scale bar for the complete sample is 500 μm, and the scale bar for the slice is 250 μm; at 25× magnification, both are 200 μm. (**B**) The transparency effect of SHANEL and iSHANEL on cynomolgus monkey brain tissue and subsequent staining with the microglia-specific marker IBA1 (purple). At 12.6× magnification, the scale bar for the complete sample is 500 μm, and the scale bar for the slice is 250 μm; at 25× magnification, both are 200 μm. (**C**) The transparency effect of SHANEL on human brain tissue and subsequent staining with the microglia-specific marker IBA1 (purple). At 12.6× magnification, the scale bar for the complete sample is 500 μm, and the scale bar for the slice is 250 μm.

Next, the effectiveness of using the iSHANEL technique to achieve transparency in large tissue samples (>1 cm^3^) was validated with pig, cynomolgus monkey. To assess the ability of iSHANEL to preserve protein immunogenicity, we conducted immunostaining on cleared brain tissue from different species using the microglia-specific marker IBA-1 (Figure 4A,B). The results demonstrated that brain tissue samples processed with iSHANEL retained high immunogenicity, enabling robust staining. Among the three species, the staining effect achieved by the SHANEL procedure was strongest in the human brain, followed by the cynomolgus monkey brain, and weakest in the pig brain (Figure 4). iSHANEL provides a valuable tool for cross-species research that enhances comparisons of the brain structures and disease mechanisms across species, offering important insights for evolutionary neurobiology and translational research.

### 2.4. A New Sample Processing Method to Reduce Blood Vessel Autofluorescence

**Figure 5:**
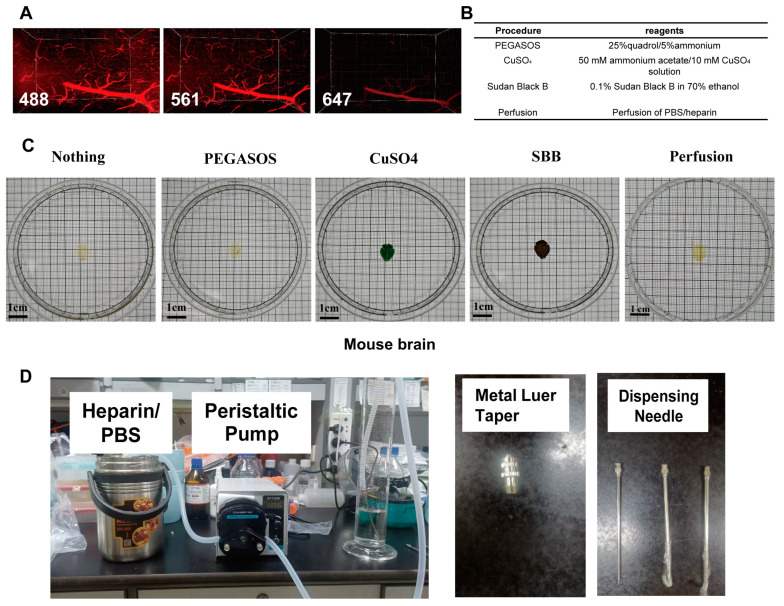

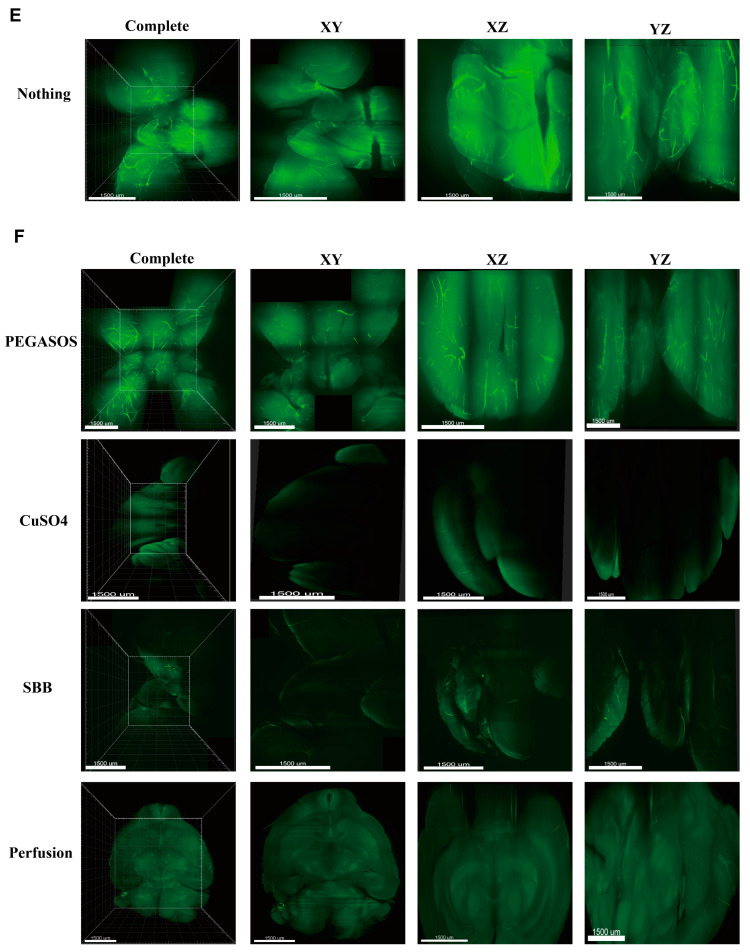
Blood vessel autofluorescence can be effectively eliminated by an active perfusion system. (**A**) The autofluorescence intensity of blood vessels in pig brain tissue under different excitation wavelengths (488 nm, 561 nm, and 647 nm). (**B**) Different methods of eliminating autofluorescence. Scale bars, 1 cm. (**C**) Comparison of the effects of different methods of eliminating autofluorescence on mouse brain tissue transparency. (**D**) Photographs of the instruments and needles used with the active perfusion system. (**E**) Spontaneous fluorescence of the mouse brain without perfusion. Scale bars, 1500 um. (**F**) Spontaneous fluorescence intensity of the mouse brain after various treatments. Scale bars, 1500 um.

Owing to the inability to perfuse most human brain tissue before clearing, heme and lipofuscin residues remain in the blood vessels of the brain, which emit strong autofluorescence in the visible light spectrum (400–700 nm), significantly hindering the tissue clearing performance and subsequent immunofluorescence imaging [19]. Initially, we compared the autofluorescence intensity in the vasculature of a nonperfused pig brain under different excitation wavelengths. The results revealed that the autofluorescence intensity was strongest under excitation at 488 nm, whereas excitation at 647 nm led to a marked reduction in autofluorescence (Figure 5A). To mitigate the impact of vascular autofluorescence, four methods for autofluorescence removal were evaluated, namely treatment with 0.1% Sudan black B (SBB) in 70% ethanol, copper sulfate treatment, the PEGASOS technique, and a novel active perfusion system using a 0.01 M phosphate-buffered saline (PBS)/heparin solution with excitation at 488 nm (Figure 5B). A perfusion system specifically for large animals was designed and successfully used on cynomolgus monkeys (Figure 5D). In the absence of perfusion, the vascular autofluorescence was highly prominent (Figure 5E). Following PEGASOS treatment, the autofluorescence remained significant. Both the copper sulfate and SBB treatments greatly reduced the autofluorescence; however, each has its own drawbacks. These three treatments resulted in incomplete observations. Additionally, copper sulfate treatment altered the tissue transparency, and, under transmitted light, only half of the mouse brain was observable (Figure 5F). Overall, perfusion not only removes the majority of autofluorescence but also preserves the original field of view without damaging the tissue structure.

## 3. Discussion

This study developed an improved SHANEL procedure, iSHANEL, which enhances tissue transparency, enabling the high-resolution 3D imaging of cleared brain tissue across multiple species. This improvement provides a more detailed view of the brain microstructures and the spatial organization of human and nonhuman primate brain tissue.

The iSHANEL method brings several improvements over existing techniques. In terms of lipid metabolism, our research reveals its efficacy in removing sphingolipids from the brain tissue of various species. Sphingolipids are major components of the myelin sheath and significantly impede tissue transparency [20,21,22]. By effectively reducing their levels, iSHANEL not only enhances the transparency but also offers insights into the lipid-related mechanisms in tissue clearing. This knowledge can be harnessed to optimize the clearing process, such as adjusting the treatment parameters for better lipid removal.

The replacement of the refractive index (RI) matching solution is another key improvement. The original SHANEL used BABB, which was corrosive and inapplicable to high-magnification imaging. iSHANEL’s use of EasyIndex overcomes these issues, allowing for 25× magnification imaging. A well-matched RI solution minimizes light scattering, which is essential for high-resolution imaging [4,19,23]. EasyIndex enables the clear visualization of fine tissue structures, like the delicate neural connections in the brain. This improvement provides more accurate morphological and structural data for histological analysis.

The broad species applicability of iSHANEL is also a major advantage. It can be used on brain tissues from nonrodents such as pigs and cynomolgus monkeys. Different species have distinct brain characteristics, and a single clearing method that works across them simplifies comparative studies [24]. For instance, it becomes possible to directly compare the brain structures and disease-related changes in different species, which is valuable for evolutionary neurobiology research.

Our exploration of decolorization methods is an important aspect of iSHANEL. The presence of heme and lipofuscin residues in brain blood vessels causes strong autofluorescence, interfering with tissue clearing and immunofluorescence imaging. By comparing different excitation wavelengths and testing methods, like treatment with 0.1% Sudan black B, copper sulfate treatment, the PEGASOS technique, and an active perfusion system, we found that perfusion is an effective way to reduce autofluorescence while preserving the tissue integrity. This optimization of the decolorization process improves the quality of the imaging results, making it easier to analyze tissue features accurately.

Despite these achievements, the iSHANEL method has limitations. It has not yet been applied to human brain tissues, and for clearing whole human brain study, we can refer to the original SHANEL study [25]. Whole-brain studies can provide a more comprehensive understanding of the brain’s structure and function. Additionally, although we have detailed the lipid removal process and lipid metabolism during delipidation, we have not explored new delipidation reagents. Developing a non-toxic delipidation reagent suitable for hydrophobic-based methods is crucial.

In summary, the iSHANEL method represents a progress in brain tissue clearing. It offers enhanced transparency, broad species compatibility, and valuable insights into lipid metabolism during clearing. However, to fully realize its potential, future research should focus on applying it to whole human brains and exploring new delipidation reagents. We also need to optimize the staining conditions to shorten the time-consuming process [26,27]. Additionally, the development of slicing devices for thick samples and light-sheet microscopes for whole samples is essential [28,29]. These efforts will help to overcome the current limitations and promote more in-depth research in the field of brain tissue studies, ultimately contributing to a better understanding of brain-related diseases.

## 4. Materials and Methods

### 4.1. Human Subjects and Animals

The study protocol was approved by the Ethics Committee of the Chinese Academy of Medical Sciences and Peking Union Medical College. Human brain samples were obtained from the National Human Brain Bank for Development and Function, Chinese Academy of Medical Sciences, and Peking Union Medical College, Beijing, China. All animal studies were approved by the Institutional Review Board of the Institute of Basic Medical Sciences, Chinese Academy of Medical Sciences. C57BL/6J male mice (8–20 weeks old) were purchased from SPF (Beijing, China) Biotechnology Co., Ltd. Mice were housed under a 12 h light/dark cycle at 23  ±  2 °C with free access to food and water. The pig brains were obtained from a local market, and the monkey brains were sourced from the Institute of Medical Biology, Chinese Academy of Medical Sciences, and Peking Union Medical College.

### 4.2. Labeling of the Vasculature

This method, similar to Professor Dan Zhu’s Ultralabel method for vascular labeling, provides an effective approach to vascular visualization [30]. The mice were deeply anesthetized with pentobarbital, and the heart was subsequently infused with 0.01 M PBS for blood washing. Gelatin solutions were prepared by dissolving porcine dermal gelatin (no. v900863; Sigma-Aldrich, St. Louis, MO, USA) in hot distilled water. Then, lysine-fixable dextran conjugated with tetramethylrhodamine (no. D1818; Thermo Fisher Scientific, Eugene, OR, USA) was dissolved in a 2% (*w*/*v*) gelatin solution to a final concentration of 0.005% (*w*/*v*), and the mixed solution was kept at 40–45 °C until infusion. Next, 10–15 mL of the mixture was infused into the mouse under warm conditions. The perfused mouse body was subsequently transferred to a low-temperature environment (approximately 4 °C) to rapidly cool and allow the gel solution in the blood vessels to solidify. After cooling, the desired mouse organs were extracted and fixed in 4% paraformaldehyde (PFA; Sigma-Aldrich, St. Louis, MO, USA, 158127) at 4 °C overnight.

### 4.3. Immunolabeling

The primary antibody used in this study was an anti-Iba1 (Wako, Japan, cat# 019-19741, dilution 1:1,000) antibody, and the secondary antibodies used were Alexa Fluor 647 goat anti-rabbit IgG (H + L) (Invitrogen, Carlsbad, CA, USA, cat# A-21245, dilution 1:500) and Alexa Fluor 488 donkey anti-rabbit IgG (H + L) (Invitrogen, Carlsbad, CA, USA, cat# A-21206, dilution 1:500) antibodies.

### 4.4. Lipidomics

Brain tissue samples from pigs, mice, and monkeys processed with iSHANEL and pig brain samples processed via various tissue clearing methods were collected and homogenized using a mixed solvent system (MTBE and methanol (MeOH) = 10:3, *v*/*v*) containing internal standards. The samples were vortexed and ground for 60 s at 50 Hz, followed by incubation on ice for 40 min. After adding water and vortexing, the samples were centrifuged at 12,000 rpm for 10 min at 4 °C. The upper layer was transferred to a new vessel, mixed with fresh solvent, and centrifuged again to concentrate the lipids. The final pellets were resuspended in a solvent mixture (isopropanol and methanol, 1:1, *v*/*v*) and transferred to liquid chromatography–mass spectrometry (LC–MS) vials for analysis.

MS was performed using electrospray ionization (ESI) in both positive and negative modes, with spray voltages of 3.5 kV and 2.4 kV, respectively. Full scans were conducted in the mass range of m/z 150–1,200 at a resolution of 60,000. Data-dependent acquisition (DDA) MS/MS experiments were performed via higher-energy collisional dissociation (HCD) at normalized collision energies of 15%, 25%, 30%, and 40% [31,32,33].

### 4.5. Comparison of the Different Tissue Clearing Methods

The whole pig brain was first fixed in 4% PFA at 4 °C for 10 days to ensure tissue preservation. After fixation, the brain was sectioned into smaller blocks with dimensions of 1 × 1 × 1 cm and 1 × 1 × 0.5 cm. The centimeter-sized pig brain samples were then cleared using the SHANEL, SHIELD, PEGASOS, Advanced CUBIC, and AKS protocols.

SHANEL [25,34]: PFA-fixed pig brain samples were washed with PBS at room temperature and then incubated with a mixed solution composed of 10% *w*/*v* 3-[(3-cholamidopropyl) dimethylammonio]-1-propanesulfonate (CHAPS) (Aladdin, Shanghai, China, C573615) and 25% *w*/*v* N-methyldiethanolamine (NMDEA; Aladdin, Shanghai, China, M105602) at 37 °C with shaking. The CHAPS/NMDEA washing procedure was repeated 2–3 times until the solution no longer turned green. Following washing with PBS for 1 day at room temperature, the brain slices were dehydrated through a graded ethanol series (50%, 70%, 100%, and 100% *v*/*v*) for 1 day and delipidated with a mixed solution of dichloromethane (DCM)/MeOH (2:1 *v*/*v*) for 1 day. Rehydration was subsequently performed using a reversed ethanol series (100%, 70%, 50%, and 0% *v*/*v*) for 1 day at room temperature. Afterward, the samples were incubated with 0.5 M acetic acid in deionized water for 1 day, followed by immersion in a mixed solution composed of 4 M guanidine hydrochloride (Sangon Biotech, Shanghai, China, A610242), 0.05 M sodium acetate (Sangon Biotech, Shanghai, China, A601611), and 2% Triton X-100 in PBS (pH = 6.0) for another day at room temperature. The slices were then briefly incubated with the 10% *w*/*v* CHAPS and 25% *w*/*v* NMDEA solution for 4 h, followed by 1 day of washing with PBS at room temperature. Finally, the samples were dehydrated again using a graded ethanol series (50%, 70%, 100%, and 100% *v*/*v*) for 1 day, delipidated with DCM (Sigma-Aldrich, St. Louis, MO, USA, 34856) for 2 days, and then incubated in a mixed solution of benzyl benzoate–benzyl alcohol (2:1), Sigma, St. Louis, MO, USA, W213802, 24122 (BABB) at room temperature until full transparency was achieved.

SHIELD [26,35]: The SHIELD solutions were purchased from LifeCanvas Technologies (Cambridge, MA, USA) and prepared according to the user guide. The samples were incubated in the SHIELD-OFF solution at 4 °C for 6 days. The samples were then transferred to the SHIELD-ON buffer and incubated at 37 °C with shaking for 24 h. Next, the samples were transferred to the SHIELD-ON buffer and incubated at 37 °C with shaking overnight. After clearing was completed, the samples were washed with PBS containing 0.02% sodium azide (PBSN) overnight. Finally, the tissue was incubated in 50% EasyIndex (LifeCanvas Technologies, Cambridge, MA, USA) with shaking at room temperature for 2 days, followed by incubation in 100% EasyIndex (refractive index 1.52) until it became transparent.

PEGASOS [36]: The samples were decolorized with 25% (*v*/*v*) N,N,N’,N’-tetraethylenediamine [2-hydroxypropyl] (Quadrol, Psaitong Biotechnology, Beijing, China, Q70015) for 2 days. The decolorized samples were sequentially incubated with 30%, 50%, and 70% (*v*/*v*) tert-butanol (Tokyo Chemical Industry, Tokyo, Japan, B0706) mixed with 3% (*w*/*v*) Quadrol for 6 h, 9 h, and 36 h, respectively. The samples were incubated with TB-PEG (containing 70% (*v*/*v*) tert-butanol, 27% (*v*/*v*) PEGMMA500 (Sigma-Aldrich, St. Louis, MO, USA, 447943), and 3% (*w*/*v*) Quadrol) for 3 days. Finally, the samples were treated with BB-PEG (containing 75% (*v*/*v*) benzyl benzoate (Sigma-Aldrich, St. Louis, MO, USA, W213802), 25% (*v*/*v*) PEGMMA500, and 3% (*w*/*v*) Quadrol) until they became transparent. All steps were performed at 37 °C with gentle shaking.

Advanced CUBIC [4]: The samples were washed overnight with PBS and subsequently immersed in CUBIC-L solution, which is a mixture of 10% (*w*/*v*) N-butyldiethanolamine (Aladdin, Shanghai, China, B106251) and 10% (*w*/*v*) Triton X-100, with gentle shaking at 45 °C for 1–2 weeks. During the delipidation process, the CUBIC-L solution was refreshed at least once. Following delipidation, the samples were washed with PBS at room temperature and immersed in a 1:1 diluted CUBIC-R solution consisting of 45% (*w*/*v*) antipyrine (Aladdin, Shanghai, China, A110659) and 30% (*w*/*v*) nicotinamide (Aladdin, Shanghai, China, N108086), with the pH optionally adjusted to 8–9 using N-butyldiethanolamine. The samples were gently shaken in the diluted solution for 1 day before being transferred to an undiluted CUBIC-R solution at room temperature, in which they were incubated until fully transparent.

AKS [37]: The samples were incubated in AKS solution until they became transparent. The AKS solution was prepared by combining 20% (*v*/*v*) dimethyl sulfoxide (DMSO), 40% (*v*/*v*) TDE (Sigma-Aldrich, St. Louis, MO, USA, 166782), 20% (*w*/*v*) sorbitol (Aladdin, Shanghai, China, S104837), and 6% (*w*/*v*, equivalent to 0.5 M) Tris base (Gentihold, Beijing, China, T1051) in ddH_2_O. The mixture was then stirred at room temperature (25 °C) until fully dissolved.

### 4.6. Elimination of Tissue Sample Autofluorescence

To eliminate the strong autofluorescence of tissue, likely caused by the presence of blood and lipofuscin, several methods were employed. The first method involved sequential treatment with 25% Quadrol for 48 h and 5% ammonium for 6 h for decolorization (PEGASOS) [36]. The second method utilized CuSO_4_, specifically a 10 mM copper sulfate and 50 mM ammonium chloride solution at pH 5.0, in which the tissue was incubated for 12 h at room temperature [25]. In the third method, 0.1% SBB was dissolved in 70% ethanol, and the samples were stained for 4 h [38]. All three methods were performed on nonperfused mice, with the treatments occurring following the second CHAPS/NMDEA protocol. The fourth method involved active perfusion, starting with 1× PBS perfusion and followed by 4% PFA perfusion.

### 4.7. Improved Tissue Clearing Method

The original SHANEL method [25] was applied to pig, monkey, and human brain tissue. However, the processing time for mouse tissue was based on the modified SHANEL method to compensate for the smaller scale and specific requirements of mouse brain clearing [39]. This method was improved as follows.

Changing the RI matching solution: Due to the corrosive nature of BABB, which can damage the lens and prevent the acquisition of high-magnification images of fine structures, BABB was replaced with EasyIndex (a hydrophilic RI matching solution, purchased from LifeCanvas Technologies, Cambridge, MA, USA, RI = 1.52). EasyIndex allows imaging at 25× magnification, ensuring clearer visualization without the risk of lens damage. This modification enhanced the imaging process, enabling safer and more detailed observations at lower magnifications.

### 4.8. Light Sheet Microscopy

The LS18 tiled light-sheet microscope (Nuohai Life Science, Shanghai, China) features advanced tiled light-sheet technology, which partially overcomes the trade-offs among the spatial resolution, imaging field of view, and optical sectioning capabilities in traditional light-sheet microscopy. It supports the imaging of samples ranging from the micrometer to centimeter scales, achieving a submicron resolution. Moreover, the LS18 microscope is compatible with various tissue clearing techniques, live imaging, and multi-color imaging. Its flexible imaging modes allow for both rapid whole-brain scanning within 10 s and high-resolution, detailed imaging.

### 4.9. Analysis Methods

The tissue samples were analyzed using the Imaris software, version 9.0.0 (ANDOR, Oxford Instruments, Oxford, UK). The data are presented as the means ± standard deviations (SDs) of three independent experiments and were analyzed using GraphPad Prism (version 9.5.0, GraphPad Software, San Diego, CA, USA. Statistical comparisons were performed using Student’s *t* test or one-way ANOVA, as appropriate. A *p* value of less than 0.05 was considered to indicate statistical significance.

## Data Availability

All research data and computer codes are available from the corresponding author upon request.

## Abbreviations

iSHANEL	Improved SHANEL
RI	Refractive index
IBA-1	Ionized Calcium-Binding Adapter Molecule 1
CuSO_4_	Cupric sulfate
SBB	Sudan Black B
CHAPS	3-[(3-cholamidopropyl) dimethylammonio]-1-propanesulfonate
NMDEA	N-methyldiethanolamine
DCM	Dichloromethane
BABB	Benzyl benzoate–benzyl alcohol (2:1)
